# Leaky Bodies and Everyday Disclosure and Exposure in the Neurodegenerative Illness Stories of Younger People With Parkinson’s Disease

**DOI:** 10.1111/1467-9566.70272

**Published:** 2026-10-01

**Authors:** Lewis Johnstone, Mark Davis, Narelle Warren

**Affiliations:** ^1^ School of Social Sciences Monash University Melbourne Australia

## Abstract

Parkinson's disease (PD) is most often diagnosed in late middle age and old age (mid to late 60s), leaving younger people with PD facing uncertainties and the long‐term challenge of life with a neurodegenerative illness. To advance understanding of the psychosocial burden associated with PD for younger people, we explored PD disclosure narratives with 22 participants diagnosed before 55 years of age. For people with neurodegenerative illness, sharing a diagnosis is only partly scripted and limited to discrete interactional events. We also consider narratives of social isolation, an impact that is not readily recognised because biomedicine mainly addresses older people with PD. The PD body discloses itself in ways that are not always controllable, nuancing sociological understanding of illness narratives to accommodate neurodegenerative illness. With reference to sociological understandings of the ‘leaky’ body and scholarship on disclosure, exposure and related perspectives on social life, we explored social expectations to explain the PD body, the avolitional, repetitive and continual qualities of these expectations and the impact of these dynamics on wellbeing.

## 1 Introduction

This paper develops a conceptual framing of ‘everyday’ illness disclosures and exposures in the lives of younger people with Parkinson's disease (PD) by showing how disclosure is not a singular event, but an ongoing, socially situated practice shaped by uncertainty, stigma and the shifting visibility of symptoms. In doing so, we advance sociological understandings of illness narrative by examining how younger people with PD negotiate the ways illness is made known in everyday life.

‘Younger’ here is relative, and refers to people who are aged 55 or under at the time of diagnosis with PD, a neurodegenerative condition typically diagnosed in older people (Demaagd and Philip 2015). The expression of PD varies considerably between individuals and over their lifetimes (Demaagd and Philip 2015; Warren et al. 2016). PD includes symptoms of involuntary rigidity, loss of spontaneous movement (akinesia), immobility (bradykinesia) and general physical limitations (Bronner 2011; Hutchinson and Raff 2000). These uncertainties and symptoms are important features of the psychosocial impact of PD and form the shifting sands upon which younger people with PD navigate their social lives.

Disclosure of diagnosis and prognosis—telling others about PD and what it might mean for the future—offers an important lens on the social impact of PD for younger people. Sharing information with loved ones, family, friends and work colleagues about any illness has implications for relationships, work, parenting and other social expectations (Pathmalingam et al. 2023; Scambler 1984; Jacobsen et al. 2022). Research on disclosure in Parkinson's disease, multiple sclerosis and other neurodegenerative conditions emphasises stigma, workplace discrimination, clinical communication and the social risks associated with revealing a diagnosis (Elliot et al. 2019; Maffoni et al. 2017; Twigg et al. 2011). However, these ways of framing disclosure are based on an assumption that it is a more or less discrete social practice. As we illustrate below, the chronic, degenerative experience of PD blurs and nuances disclosure in ways that are important to understand for effective care and support. Jutel and Nettleton (2011) called for a sociology of diagnosis that includes biographical and temporal consequences. Our research responds to that call in light of the lived experience of younger people with PD. In what follows, we provide further explanation of the social framing of diagnosis and prognosis we depend on in this article and, in particular, what is meant by the everyday disclosures linked with PD. We also explain the specific implications of PD for younger people and how they intersect with social life.

## 2 Background

### 2.1 Theoretical Framing

Examining the lived experience of PD advances sociological framings of diagnosis, disclosure and illness narrative more generally by drawing attention to disclosure of illness as an embodied and potentially involuntary process. As we argue, because of the ongoing, changeable and increasing symptoms of PD, people with the condition are often exposed in ways that create telling imperatives. Symptoms observed in everyday life, for example, going to the shops, managing pain, fixing lunch, exhibiting tiredness, timing medications and other everyday experiences can lead to questions from family, friends and strangers. Questions such as ‘Are you OK?’ or ‘How are you today?’ place a potential duty of disclosure on the person with PD, situations that are difficult to control because of symptoms that are not easy to curb and conceal (Warren et al. 2016). In this way, the PD body tells in ways that interrupt the self and social relations, generating a repeated need to explain, disclose and repair the meanings attached to visible symptoms. Goffman's notion of self‐presentation in everyday life (Goffman 1959; Crossley 2022) is apt for reckoning with the social impacts of PD disclosures and exposures, as it shows how social interactions depend on management of appearances and disclosures. PD, however, complicates this management as the body may offer signs of illness involuntarily, requiring people to explain, repair or reframe symptoms that have been noticed. Moreover, we adapt Goffman's account of stigma by shifting attention from the management of what is known or concealed to emphasise the embodied and recurring ways illness becomes visible. For people with PD, disclosure is not confined to the intentional narration of diagnosis. Instead, symptoms convey information, invite interpretation and require ongoing acts of explanation and repair throughout the course of a long‐term degenerative neurological illness.

In this sense, recurrent, involuntary PD disclosures and exposures leak into social worlds. To develop this argument, we bring Goffman into conversation with Shildrick's feminist theorisation of the ‘leaky’ body. Rather than extending these frameworks, we use both in tandem to display disclosure as a co‐produced, neither wholly chosen nor wholly imposed, practice. Feminist scholarship on the body in biomedicine has used the leaky body to challenge normative, gendered assumptions of the body as bounded, stable and self‐contained (Shildrick 1997). The leaky body helps to explore the biopolitics of blood, reproductive fluids and other forms of embodiment that matter in biological life and lived experience. Leaky body framings have been used in research on menopause (Whiley et al. 2023), women's reproductive health (Shildrick 2015), public health (Charnley 2021) and disability studies (Remnant et al. 2023). In this paper, we extend this metaphor of leaky embodiment to help examine how PD symptoms leak into social life and disrupt body norms, identity and social interaction, creating scenarios and demands to repair these bodily transgressions. Read together, Goffman and Shildrick's concepts show how PD disclosure is an embodied, relational and recurring process rather than an individual, one‐off decision to reveal a diagnosis. As we consider in what follows, PD disclosures are specifically repetitive and unfamiliar as participants live with, in and through their increasingly impaired, leaky bodies.

#### 2.1.1 Disclosing PD Diagnosis and Prognosis

The disclosure to others of information about one's health provides an important lens on PD and its ramifications for social experience. Social relationships are shaped through the exchange of information about health and can take various forms. For example, disclosure may be required to access leave of absence from the workplace (Hastuti and Timming 2021). In research settings and medical record keeping, patients may be asked to provide information and consent to its use by third parties (Tapuria et al. 2021). Sharing information with a medical practitioner to help them make a diagnosis and select a treatment can also be seen as a form of collaborative disclosure (Macduffie et al. 2021).

Health‐related disclosures often take the form of illness narratives that situate illness in its social context and establish the narrator‐listener relation (Andrews et al. 2025). The orthodox narrative of illness followed by recovery is the canonical narrative of medicine. Individuals are expected to dutifully subscribe to expert care, recover and return to normality, and disclosures help individuals to conduct themselves in these ways (Monaghan and Bury 2022). PD, however, transgresses this expected narrative because recovery is not possible. For this reason, narrating a PD diagnosis is imbued with heavy connotations of loss and decay, which can trigger stigma and social exclusion (Crooks et al. 2025; Hanff et al. 2022; Mcdaniels et al. 2023). In this sense, stigma is understood here as a relational process through which visible symptoms, diagnostic labels and associations with decline mark people as socially diminished or out of step with expectations of independence.

Sharing information about PD may also mobilise social control in the form of the surveillance of conduct, by self and others. Disclosing PD with an outwardly healthy body can also be difficult because it tells of future degeneration, particularly for younger people. Disclosure of PD diagnosis, then, can place the individual outside of normative temporal ordering of their biography, setting them apart as not normal (Scambler 2009). This othered status can sponsor pity, disgust or tragedy for those diagnosed with PD. Concealing and minimising symptoms may become important to produce a normal, unimpeded body and maintain normative personhood (Davis and Manderson 2014; Li and Lee 2023). Finding ways of resisting these narratives of loss, stigma and disablement is, as we will see, an important life strategy for people with PD.

#### 2.1.2 ‘Everyday’ PD Disclosures and Exposures

As we indicated previously, PD is particular in that disclosure, or exposure, can be imposed by the PD body and, because the condition is chronic and degenerative, disclosure and exposure are incessant and changeable. We use the double meaning of ‘everyday’ to emphasise the repetitive and quotidian features of disclosure and exposure for people with PD. We also link disclosure and exposure with Goffman's dramaturgical theory of self and everyday life (Goffman 1959) and related scholarship (Crossley 2022). Revealing oneself to others is entwined with self‐identity and the social relations of day‐to‐day social interaction. According to dramaturgy (Goffman 1959), disclosure acts reveal how the narrator expects to be seen by others, often with recourse to pre‐existing assumptions about individuals, their social standing and links with others. As we have noted, the PD body is not always and wholly controllable, which means that disclosure, exposure and related practices of personal experience narration sometimes force the person to improvise on the spot. Goffman's dramaturgical metaphor also helps to explain how disclosures and explanations after exposure might be shaped to avoid stigma and prejudice. We also adopt the view that sharing a PD lived experience is partial in the sense that language practices and interactional life structure constrain what can be made known about life with PD (J. Butler 2005).

#### 2.1.3 Younger People and PD Diagnosis

Everyday disclosure of diagnosis has particular implications for younger people with PD. The mean age of PD diagnosis in Australia is mid‐60s, and approximately 20% are diagnosed prior to their 50s (Bivol et al. 2022). There is no blood test for PD, so diagnosis is surmised by symptoms, age and reaction to pharmaceutical treatment (Warren et al. 2016), which can leave younger people uncertain about their diagnosis for a considerable period of time. In addition, prognosis for someone diagnosed with PD is uncertain and, particularly for younger people, can compound the challenges of work, parenting and social life. Younger people with PD may not be certain about what they should say about their diagnosis and prognosis because of these uncertainties.

Younger people with PD may also face unwanted, pre‐emptive medicalisation. In medical discourse, the acronym YOPD (young‐onset Parkinson's disease) is used to distinguish between younger and older people (Mehanna et al. 2022). YOPD is a somewhat medicalised and ageist term that reduces individuals to biographical and neurological characteristics, another dimension of the social burden of the condition. Younger people with PD may be positioned as having a medical problem—a disease—rather than a problem of social interpretation—illness and disability (Nijhof 1995). This conflation of medical issues obscures the social impacts that PD has on younger people and contributes to delays in receiving formal support services, which are often adapted from services designed for older adults with PD (Stephenson et al. 2023). In this paper, we prefer the term ‘younger people with PD’ as less reductive terminology.

Because PD in younger people is diagnosed in socially and economically active stages of life, the psychosocial burdens experienced by younger adults with PD are specific. Parenting, employment, relationships and sexuality are all strained, and the support required to navigate these burdens can be difficult to access (Stephenson et al. 2023). It is also known that people with PD have been found to exhibit high levels of depression and anxiety (Mehanna and Jankovic 2019), though these considerations for younger people with PD have not been researched specifically. Obtaining a PD diagnosis at a younger age is likely to have specific considerations, including taking on an identity of bodily change and loss typically linked with older people.

Therefore, within this article we explore ‘everyday’ PD disclosures in relation to narrative agency, the leaky body and degenerating out of normal time. We pursue insights that move beyond normative, cognitive notions of illness disclosure and narrative. We explore how the embodied self, living with PD, enacts disclosure and responds to exposure in interactional life. We also consider the ongoing, changeable disclosures and exposures and the impact on the lived experience narratives and temporalities of those with PD. By exploring how participants tell of the body as it repeatedly becomes unfamiliar and transgressive, this article generates new insights into the social impact of living with PD at a younger age and related implications for the sociology of illness narrative.

## 3 Methods

We used qualitative interviews and photovoice methods informed by illness narrative to explore how younger people with PD experienced telling others about diagnosis and prognosis. Participants were selected according to these purposive criteria: having obtained a medical diagnosis of PD before the age of 55 years; an even mix of gender identities. Participants were recruited through Parkinson's organisations, social media pages and neurology clinics, where we were granted access to invite research participants through flyers. The first author interviewed 22 people with PD residing in Victoria (*n* = 13), New South Wales (*n* = 8) and Tasmania (*n* = 1). This geographical distribution is relevant because experiences of disclosure may be shaped by local worlds, including access to specialist care, workplace arrangements and the degree of anonymity available in urban or regional settings. However, the study was not designed as a comparative analysis of location. We therefore treat location as part of the participants' lived context rather than as a variable for generalisable claims.

Eleven participants reported that they identified as men and 11 as women. The participants were aged between 36 and 67 years. The years since diagnosis (at the time of first interview) ranged from 6 months to 20 years. In‐depth interviews were conducted with all 22 participants, and 13 of these people participated in the photovoice activity and a follow‐up interview to provide greater insight into the lived experience of those diagnosed with PD before 55 years of age. Interviewees provided written informed consent (Monash University Human Research Ethics Committee ID: 31721).

A semi‐structured interview guide was developed to ensure consistency while allowing participants to elaborate on meaningful experiences (Kallio et al. 2016). This guide focused on symptom onset, diagnosis, everyday life and disclosure. This approach enabled participants to narrate disclosure in relation to biographical and social contexts.

Photovoice methods were used to complement the in‐depth interviews, helping to explore the social environments of the participants' local worlds, their related meanings and their experiential narrative. 6 months after the initial interview (except for one participant who requested to take part in the photovoice activity 3 months after the initial interview), we invited all 22 participants to create photographs or short videos of meaningful experiences. We asked them to do this each day over a 2‐week period. Participants were then asked to take part in a follow‐up in‐depth interview, in which their photos were discussed. Fifteen participants returned photographs, and 13 participants took part in a follow‐up interview. The photovoice activity added subtle nuances, metaphors and visual elements to the participants' narratives—elements that narrative interviews alone would not have produced.

The transcribed interviews and photovoice materials were analysed using an experience‐centred narrative analysis (Andrews et al. 2013). We employed this approach to align with the aims of illuminating an under‐researched phenomenon and to explore narratives on the lived realities of social worlds (Squire, Andrews, et al. 2014). This approach is an appropriate choice for analysing chronic illness narratives, which are often marked by specific biographical markers or events and experiences, such as disclosure, that disrupt, change or shift narratives and require re‐organising or reconstruction of selves through a process of meaning‐making.

The interview and photovoice materials were analysed using an inductive, constant comparison method (Corbin and Strauss 2015). Contextual influences were interpreted as part of lived experience, ensuring findings remained grounded in participants' social worlds (Squire, Davis, et al. 2014). As one of the aims of this research was to gain a deep understanding of the dynamic process (Salkind 2010) of living with PD, we chose to localise knowledge to analyse the participants' lived experience rather than make inferences about all people with PD. Verbatim transcripts and images were first read and examined in an open‐ended way. This immersive approach was an attempt to reflect on the participants’ experiences and feelings expressed in what was being said (Corbin and Strauss 2015). Second, the materials were then examined in greater detail and coded inductively to generate themes to address the research aim. Codes were compared across participants to identify patterns and differences in everyday disclosure, while attending to age, gender and family responsibilities. All interview fragments are attributed to a pseudonym.

## 4 Findings

We present our findings with reference to four themes that address our research focus on illness disclosure among young people with PD: the body requires explanation; disclosure and exposure leakages; scripting disclosure and shielding others from the everydayness of PD.

### 4.1 The Body Requires Explanation

For our interviewees, disclosure was not a one‐off event but a repeated practice, as everyday encounters with others and their unfamiliar body continually demanded explanation. For example, Eric, a 37‐year‐old man who was diagnosed approximately 3 years prior to his interview, discusses everyday encounters as he moved about the city in which he resided.

I would smile at people in lifts or passing them and people would have a weird reaction where they weren't sure what was going on with me. People would check in and say, “Is everything all right? You look a bit upset.” And I would have to explain, “It’s just Parkinson’s.”

(First interview)

Eric is speaking of hypomimia, also known as the ‘Parkinson's mask’, a reduction of spontaneous facial movement or emotional facial expressions (Bianchini et al. 2024). His symptoms create the need for explanation and therefore disclosure. It is an instance of micro‐disclosure as it is both typical and recurrent as Eric interacts with people during daily life. Eric's statement, ‘I would have to explain, “It's just Parkinson's”,’ indicates that a PD body causes a necessity to tell of chronic illness (Manderson 2014) and justify itself in relation to social norms, such as smiling. Eric shrugged his shoulders when providing these examples, intimating resignation towards these social interactions and demands for explanation. In contrast, Robin, a 52‐year‐old woman diagnosed at 32 years of age, discusses her frustration with those who demand explanation.

When I did tell people and after DBS [deep brain stimulation], because I had all my head shaved. So now I have this bald head. And I thought I'm going to go out and wear my bald head with pride. Scars and all. So, I'm walking down the street and the people I found could be divided into three groups; people that would say, “Oh, what happened to you?” Then you get the idiots, “Oh, you poor thing”, like I was stupid or something. And then nasty people that are downright rude, who would say, “You shouldn't be out”. I'm thinking well, “Fuck you”.

Although Robin has been living with YOPD for approximately 20 years and much longer than Eric, who is considered to be at an early stage of his neurodegenerative illness narrative, they both describe how the PD body's appearance attracts attention and necessitates explanation and truth‐telling (Manderson 2014). Robin's account shows how visible departures from social norms produce interactional friction, as she uses reported speech to retell how PD is narrated and repeatedly negotiated in everyday life. Notable is Robin's explanation of how her illness narrative could be framed by prevailing assumptions of chronic illness, disability and tragedy. Although Robin resists the active stigma that her appearance post‐DBS attracts by wearing her ‘bald head with pride. Scars and all’, neurodegenerative illness narratives are often interpreted as a relentless decline in physical and cognitive ability and persistent pain, a representation that helps to mobilise pity (or stronger emotions like disgust) towards those with a disability or, worse, generates a social refusal to acknowledge the PD body altogether (Seddon et al. 2018). Robin speaks of the stigma reflected in the speech of others, ‘You poor thing’ (pity) and ‘You shouldn't be out’ (disgust) and underlines the repetitive nature of everyday disclosures, so much so that she is able to categorise different kinds of responses to her post‐treatment body and her diagnosis.

These accounts should not be read as narratives of social diminishment. Instead, they show how participants face everyday dilemmas of disclosure and how they actively work with meanings attached to PD, as they learn to explain it, when to resist explanation or selectively disclose information. Importantly, the temporal aspects of PD are significant here too. This is in part because an unpredictable prognosis means that disclosure continues to unfold alongside a changing condition. Disclosure is therefore not only repeated because PD is chronic but because the body changes across time, generating new moments in which symptoms, treatments and appearances require explanation and in which individuals use disclosure to manage their experience of PD.

Whereas Eric and Robin described examples of justifying the body in public spaces, instances of everyday disclosure also occur in private spaces. Belinda, a 61‐year‐old woman, who was diagnosed 18 years prior to her first interview, lived with her husband and one of her adult children. In this fragment, she describes an instance of everyday disclosure.

Interviewer Has Dale (husband) working from home been a change to your relationship?

Belinda Well only when he comes out at lunchtime asking what's for lunch (both laugh). My son's home a lot now too and every time a noise goes off, like if I drop something, somebody always says, ‘Are you okay?’ And these days if I drop something I yell out, ‘I'm okay.’ Because I know what's going to happen.

Belinda's example highlights how the noises she makes may require explanation. These instances are reminiscent of disclosures in the sense that others require reassurance for their own sense that something may be wrong. Belinda is not asked to explain her diagnosis in a literal sense, but is asked to provide information and repair the social order. Notable here, too, is that Belinda's body is out of sight, but the sound it produces still requires explanation, a perspective that suggests a family imaginary of the PD body and its degeneration.

### 4.2 Disclosure and Exposure Leakages

Disclosure is often thought of as a momentous and specific interaction, leading to a changed body and sense of self in relation to others (Li and Lee 2023). As Eric and Robin suggest, disclosure can also be demanded by others in typical and repeated situations. In other scenarios, however, research participants highlighted how disclosure leaked in ways that bracketed aside their agency. In these situations, the PD body and its changes exposed the individual and created the need to explain themselves and reassure others. Disclosure in this view was embodied and visceral and therefore not simply a pre‐coded, agential narration of illness.

An illustrative example comes from Nancy, a 67‐year‐old woman, diagnosed at 50, who spoke of increasingly disclosing her illness as her symptoms progressed, and how these changes and social expectations impact her self‐worth. When asked if she has ever felt or seen herself as different since diagnosis, Nancy responded:

Nancy I don’t see it as much as other people comment on it because I get used to it. Whereas other people who may not have seen me for a while will say things like, ‘I can see you’re getting worse.’ Which is really nice (sarcasm).

Interviewer How does that make you feel?

Nancy Well, it makes you feel worse. It makes you feel I must be getting worse because they’ve noticed. Like a cousin’s child came up to me at a family get‐together. And they said to me, ‘Why are you always shaking?’ He’s about six, and his mother said, ‘She cannot help it. It’s something that she can’t help.’

Nancy’s story exhibits both the typical and recurrent aspects of the everydayness of disclosure when living with PD at a younger age. The child's remarks about her body suggest a body out of control, and the mother of the child confirms this: ‘she cannot help it’. Nancy implies in her story that the mother is aware of her diagnosis and discloses on her behalf, nuancing the sense in which leaky disclosures undermine personal control. Nancy's example suggests that the orthodox conceptualisation of disclosure commonly applied to other illnesses is too cognitive and agential to be helpful for the PD experience. The metaphor of ‘leakage’ better captures what happens on an everyday basis because it captures the incidental and co‐produced PD quality of exposure in social settings. Leakage is ontologically complex because it occurs at the confluence of multiple factors: Nancy’s shaky body after a workout, a last‐minute invite to a barbeque, the directness of a child’s remark that marks this leakage and the final comment in the third person, ‘… she cannot help it.’ Nancy’s and others’ experience of leakage presents a need to justify the body that is out of control (J. A. Butler and Modaff 2016), repeatedly and continually. Nancy’s story further complicates our notion of disclosure and continues to raise, rather firmly, essential questions of who is served by disclosure. In her story, disclosure is imposed and stands as an explanation for the child. Nancy’s story is a cause to reflect on how people with PD can be assisted to manage deliberate and incidental disclosures and exposures, not only for those disclosing but for those for whom the disclosure is made.

Nancy’s experiential narrative suggests how a body can tell on itself, an experience that was a common theme in the interviews. Interviewees spoke of strategically keeping their diagnosis invisible, but at times having to justify the appearance of their bodies to others. They were compelled to create a viable story that avoided revealing their chronic illness. For example, Hannah discusses day‐to‐day life with PD with reference to how her body revealed itself. Hannah reported that she would hide PD by claiming that her constrained movements were due to a sore back. However, as Hannah explains, as symptoms increasingly emerged and impinged on daily life, invisibility became untenable.

Hannah I chose that if people asked, ‘You got a sore back?’ Yeah, I just got a bit of a sore back. So, I played it off for a while. Now I’m like, ‘You know what, it’s not getting any better’. It’s not going to get any better. So, I might be straight up and say, ‘I've got Parkinson’s’. If I’m unsteady at some point, or my meds are not working, I say it straight out now because it’s easier. You still get the looks, but at least they understand. It gives them a reason to know why you can’t move out of their way or why you look like you're drunk when you’re not. So, for me, I find it easiest to tell them rather than get the looks like you’re a weirdo. I started saying to anybody and everybody because they’d be looking over and be like, ‘Excuse me, can you move?’ and I’m like, ‘I'm really sorry, I can’t right now. I’ve got Parkinson’s, so I'm kind of stuck at the moment’… It almost feels like instead of going, ‘Hi, I’m Hannah.,’ it’s almost like; ‘Hi I’m Hannah, I’ve got Parkinson’s.’ I shouldn’t have to do that but at the same time I feel very compelled to explain because of what I couldn’t do, and that was happening all the time.

In this fragment, Hannah explains how the degenerating PD body creates a necessity to disclose her diagnosis. Hannah recalls that, for a time, it was possible not to share her PD diagnosis, but that increasingly the questioning glances of strangers gave her a sense that her body signified deviance, such as looking drunk, unsteady on her feet or a ‘weirdo’ not in control of her body. Hannah, then, trades off one kind of exposure for another. She shares her diagnosis and its connotations to mitigate the judging stares of strangers. Hannah makes clear the pressure upon those with PD to repeatedly disclose their diagnosis as they interact with others, but also a reluctant acceptance of PD self‐identity. Significantly, Hannah indicates that all of her choices—invisibility, unexplained symptoms, PD disclosure—involve different combinations of benefits and risks to self‐identity and relations with others.

### 4.3 Scripting Disclosure

The pressures to disclose illness and explain exposures have consequences for wellbeing. Hannah and Robin display frustration at the need to disclose their diagnosis repeatedly. This is perhaps in part because of the physical, social and emotional energy that is required to disclose. Disclosing illness is often depicted as a staged process that is rehearsed and performed (Woodgate et al. 2022), which implies that it takes time and energy. Wendy, a 49‐year‐old woman who was diagnosed less than 12 months prior to the interview, captures this rehearsal process and explains how each time she performs disclosure it is exhausting, as it requires managing others’ feelings.

Wendy You feel bad when you tell people because I guess it’s a lot, and it’s not a 10‐min conversation. You can’t say, ‘oh, by the way, I’ve got Parkinson’s.’ You’ve got to go through the whole process so they can get their head around it, and then they’ve got questions. So, every time you tell someone, it’s quite exhausting. You’re kind of having to reassure people that, ‘I'm okay, I’m still the same, I’m managing it.’

Wendy notes that disclosing PD is emotionally and physically taxing as it requires answering medical, clinical, treatment and cure questions and also explaining prognosis. Part of what is so taxing here is the recurrent revisiting of an uncertain future with PD. Wendy, similar to several other participants, discusses a need to manage the listener’s emotions and reassure them that the management of the disease is going as best as possible. Wendy’s account of disclosure performance creates imagery of getting into character, finding the right setting, putting on a brave face or a mask, and anticipating reactions, emotions and questions. Moreover, this performance needs to be called upon repeatedly and adjusted time and time again depending on for whom the performance is made.

For others, though, scripting disclosure proved challenging because PD is itself difficult to explain. Thomas, a 49‐year‐old man, diagnosed only 6 months prior to his initial interview, had only disclosed his PD diagnosis to his wife and daughter. He discussed why he has not told members of his social network through an example of meeting close friends at a bar.

Thomas I have a group of friends that we've all known each other since fifth grade. So, we all got together at the local pub. And I kept information about what was happening in my life to a minimum. I kept asking questions, rather than trying to answer questions, and I left like an hour or so in. Because I didn't want to be around

Interviewer Why do you think you haven’t wanted to tell this group of friends?

Thomas I think it’s more because I don’t understand it [PD] myself. So, trying to explain what Parkinson's is might be really difficult. I think I’m a bit embarrassed by it. But I’m not sure, I don’t feel comfortable saying it

As Wendy's example shows, disclosing requires a specific and significant amount of knowledge, as listeners often have an emotional response that requires some form of empathetic or considered response from the discloser, along with being able to answer questions about the illness. Similarly, Thomas's experience indicates that his knowledge about PD shapes his capacity to disclose. His recent diagnosis and limited understanding of PD make him unwilling to disclose. Thomas's suggestion of embarrassment and discomfort also displays how disclosure involves more than relaying medical information. It requires finding socially acceptable ways to explain a changing body and uncertain future. Thomas's example, like others in our research, raises questions of who is served by disclosure.

### 4.4 Shielding Others From the Everydayness of PD

The social significance of PD disclosure is also apparent in the interviews when participants spoke of shielding others from the day‐to‐day management of symptoms. In her interview, Wendy described PD as a ‘moving beast’ entailing fluctuating symptoms without any link to volitional behaviour (Bramley and Eatough 2005). Participants described how they would eat well, get proper sleep and exercise to help manage the daily impact of PD and yet good days could turn to bad days within minutes. Peter, a 51‐year‐old man who was diagnosed approximately 3 years prior to our meeting and worked as a musician, was in a de facto relationship with someone and had three children under 16 years of age. He raised the need for emotional and physical support.

Interviewer So, you mean like someone that knows how you feel? Another person with Parkinson’s, perhaps at a similar stage or similar demographic?

Peter Yeah, I’m talking more about professional Parkinson’s nurse or a support worker that’s got Parkinson’s or knows a bit about it. The ins and outs of what it can bring, what it can do to you. Because there’s a lot of stuff that goes on behind the surface with Parkinson’s that people can’t see. So, when you’re feeling a particular way, a lot of people can’t tell. My wife, for example, she can’t really tell if I’m feeling that crap because it’s kind of in there [Peter points to his chest], you know? It would be nice to have someone to talk to about that

Interviewer Is that something that you try and keep from your wife? When you are kind of feeling crap, and it’s internal? Is that why you’re saying having one of those professionals available would be nice?

Peter Yeah, because there’s no point laying it on my wife because she doesn’t know what it feels like, there’s no point. I don’t know if there’s any point and trying to make her understand how it feels, and I don’t want to lay this on her a lot of the time. But I’m feeling crap and unless it’s completely obvious to her, which occasionally she does say you look a bit off today, I don’t tend to mention it very often. And we really don’t talk about my condition very often in a regular week. It wouldn’t come up normally at all. We don’t really talk about it very often

Peter reveals that PD is not just a physical battle. It has emotional impacts, as he asks questions about the future of what PD ‘brings … what it can do to you?’ He expresses the view that the impact of PD occurs beyond what others can see, which has been described by interviewees as ‘internal shaking’ (George, aged 60 and diagnosed at 53) and ‘vibrating’ (Cameron, aged 41 and diagnosed at 34). Participants also described how the internal aspects of PD were challenging to communicate, especially to a partner or family member, not only because of the challenges in describing the sensation but also because of the perceived burden this may place upon the listener. Echoing Wendy's scripting of disclosure discussed above, decisions about what to tell become important. Peter’s final comments from this excerpt capture this disclosure challenge, as he struggles to find the benefit of repeatedly telling his wife, as he notes, ‘I'm feeling crap and unless it’s completely obvious to her…I don’t tend to mention it very often.’

Another way to shield others from PD was to live alone. Fiona, a 38‐year‐old woman who had been experiencing symptoms since she was 32 years of age and diagnosed at 34, lives near her family (within walking distance), who have offered to have her stay with them. However, she prefers to live alone to manage her PD. Over the course of the interview, she reveals how living alone is a way to avoid the repeated need to explain her ‘moving beast’ (Wendy, quoted above). In this fragment, Fiona describes a situation similar to Peter's but makes a connection to her sense of personhood.

Interviewer Do you think imagining a future with Parkinson’s perhaps influences what you do in the present?

Fiona Yeah, I think it really does. For me, I’ve always been reasonably high functioning and intelligent and capable physically. I’m lucky things have never been terribly hard. I’ve been able to learn and understand quickly. But knowing that and being able to now feel and see, not just me, but other people, my symptoms are sort of becoming noticeable externally. And I think that’s the biggest thing for me, I’m quite conscious of trying to protect everybody from seeing the bad side of this (PD). And I’m already doing that. I don’t want other people feeling awful … … And I guess I’ve already been trying to protect people from that. Like, it’s no secret, my diagnosis. I’m quite open about it in terms of what it actually does to me. But I’m conscious of protecting others from it

Fiona discusses how symptoms impact her perception of herself pre‐ and post‐PD. She shields her family and friends from witnessing the change from a high‐functioning individual to no longer being the same person. Fiona describes how she does not want others ‘feeling awful.’ By ‘protecting’ those around her from seeing these changes, she avoids everyday instances of disclosure and exposure. For Fiona, shielding also helps to maintain a sense of internal or self‐perceived normality and personhood, and a continuation of a previously envisioned life story. PD is degenerative, and participants’ bodies and sense of self change in many ways. Shielding acts as a way to resist the changing body/self and changes to personhood through time.

## 5 Discussion

Our findings suggest that for younger people with PD, the ontological ramifications of disclosure are not confined to the clinic and are also linked with exposures and other features of the leaky PD body. For people with PD, disclosure (re)surfaces in the repetitive, incidental disclosures demanded by a changing body. We described these encounters as micro‐disclosures, in which everyday moments of illness become socially legible and require explanation. This article, therefore, contributes to a sociology of illness by foregrounding the temporal, spatial and embodied dynamics of disclosure in everyday life. By examining how participants rehearse, perform, hide and continuously negotiate their identities across public and private spaces, this research contributes to an understanding of disclosure as an iterative and relational practice shaped by bodily unpredictability, social expectations and the labour of managing what others see. In doing so, it reframes the disclosure of neurological conditions not as a discrete moment, but as an everyday, repetitive and often incidental process that is inseparable from the body's visibility, the organisation of social space and the temporalities of chronic illness.

This contributes to a broader sociology of illnesses that occur earlier in the life course than culturally anticipated. For example, research on younger people with arthritis shows how age‐based assumptions shape whether symptoms are recognised, whereas young‐onset dementia research identifies disclosure as a means of explaining behaviour, accessing support and challenging age‐related stigma (Kirkpatrick et al. 2018; Kohl et al. 2024). These studies demonstrate that illness is experienced not only through pathology but through its timing within the life course. Our findings extend this literature by showing that age also shapes the interactional interpretation of symptoms. When illness is not anticipated in younger bodies, symptoms may not be recognised as illness, but interpreted as incompetence or abnormal behaviour, intensifying the expectation to make the body intelligible to others. This moves sociological understandings of disclosure beyond models that privilege conscious and rehearsed decisions to reveal or conceal diagnostic information (Kaushansky et al. 2017; Manderson et al. 2015; Woodgate et al. 2022). Participants' accounts of ‘having to explain’ their bodies in lifts, streets, homes, workplaces and family gatherings illustrate the extent to which PD continually requires clarification.

The body‐that‐requires‐explanation showed how hypomimia, tremor, slowness and dropping objects unsettle interactional expectations and invited queries such as ‘Are you okay?’ or ‘What's wrong with you?’ In these moments, participants were positioned as ‘truth‐tellers’ of their condition (Manderson 2014, 2), compelled to narrate PD to repair a disrupted social order, even when they might prefer not to. Disclosure here functions less as a planned performance but more as a form of social maintenance in which the person with PD must repeatedly justify a body that does not conform to norms of expression or control.

This also helps explain why the psychosocial burden of disclosure can accumulate. The significance of micro‐disclosures lies less in any single encounter than in their repetition across public and private spaces. Participants' scripting and shielding practices show that disclosure involves not only explaining PD but anticipating and managing others' responses. Shielding partners, children or friends from symptom severity, minimising discussion of PD or withdrawing from situations in which explanation may be required can reduce immediate interactional demands while narrowing social participation over time. This resonates with young‐onset dementia research showing that misunderstanding and stigmatising responses can contribute to shrinking social networks (Kohl et al. 2024). Our findings add a mechanism through which such isolation may develop, as social withdrawal can emerge cumulatively from the exhaustion of having a changing body repeatedly noticed, misread and explained.

The concepts of leakage and the body‐that‐requires‐explanation help account for this work. In Goffmanian terms, participants engaged in the management of self‐presentation, scripting disclosure, selecting audiences, concealing diagnosis and repairing unwanted interpretations (Goffman 1959). Yet Shildrick (2015) exposes the limits of understanding these practices primarily through intentional self‐presentation. Although Shildrick's account emerges from feminist critiques of gendered ideals of bodily boundedness, she shows how expectations of containment and control operate across bodies. Tremor, altered expression, medication fluctuations or treatment effects can leak information before the individual has decided what to say. Read together, Goffman and Shildrick therefore allow disclosure to be understood as co‐produced, neither wholly chosen nor wholly imposed, but negotiated at the intersection of embodied change and social interpretation.

Responding to Jutel and Nettleton's (2011) call for a sociology of diagnosis that includes biographical and temporal consequences, our findings show how a diagnosis of Parkinson's for younger people continues to have a presence in everyday interactions that create instances of micro‐disclosures. These micro‐disclosures show how illnesses (re)surface or leak through the body as symptoms change and become meaningful to others. These themes suggest that disclosure for younger people with PD is better understood as an ongoing, situated practice of managing visibility, explanation and moral responsibility, as opposed to a mise‐en‐scene‐like, discrete event. The double sense of everyday—as both repetitive and quotidian—is central here. Because PD is chronic, degenerative and clinically uncertain, and because its symptoms are variably expressed in and through the body and its surroundings, demands for disclosure recur across the day and over the life course. Our analysis complicates understanding of disclosure as a one‐off ‘coming out’. Although such disclosures can be significant, they are only one part of a much broader array of everyday disclosures that are required continuously and repetitively.

## 6 Conclusion

These insights have implications for the sociology of illness narrative and for work on health‐related disclosure more broadly. First, they underscore the need for theoretical accounts of disclosure that accommodate temporality and degenerative corporeality. Rather than positioning disclosure as a rational, one‐off act of information transfer, our findings argue for a conceptualisation of disclosure as ongoing and incidental, shaped by the unpredictability of bodily symptoms, the organisation of space and the demands of relationships. Second, by centring younger people with PD, the study extends existing research that has largely focused on older populations or on discrete domains such as employment or clinical communication (Maffoni et al. 2017; Twigg et al. 2011). It highlights how life stage and prognostic uncertainty shape disclosure practices and suggests that age and temporality should be incorporated into analyses of chronic illness narrative.

Finally, these findings suggest practical implications for care and support. Health professionals might better recognise the emotional and interactional labour involved in everyday disclosure and create space in consultations to discuss not only whether patients have ‘told others’, but also how they are navigating recurrent, incidental and imposed disclosures. In sum, by foregrounding everyday disclosures, this study contributes conceptual and empirical nuance to understandings of disclosure in neurological conditions and extends social theory on illness narrative, embodiment and time.

## Author Contributions

**Lewis Johnstone:** conceptualization, investigation, writing – original draft, methodology, writing – review and editing, formal analysis. **Mark Davis:** writing – review and editing, writing – original draft, conceptualization, methodology. **Narelle Warren:** writing – review and editing, writing – original draft, conceptualization, methodology.

## Funding

The authors have nothing to report.

## Conflicts of Interest

The authors declare no conflicts of interest.

## Data Availability

Research data are not shared.
